# Bio-Polyethylene Composites Based on Sugar Cane and Curauá Fiber: An Experimental Study

**DOI:** 10.3390/polym15061369

**Published:** 2023-03-09

**Authors:** Gustavo Henrique de Almeida Barbalho, José Jefferson da Silva Nascimento, Lucineide Balbino da Silva, Ricardo Soares Gomez, Daniel Oliveira de Farias, Diego David Silva Diniz, Rosilda Sousa Santos, Maria José de Figueiredo, Antonio Gilson Barbosa de Lima

**Affiliations:** 1 Federal Institute of Education, Science and Technology of Rio Grande do Norte, Canguaretama 58190-000, Rio Grande do Norte, Brazil; 2 Department of Materials Engineering, Federal University of Campina Grande, Campina Grande 58429-900, Paraiba, Brazil; 3 Department of Materials Engineering, Federal University of Paraiba, João Pessoa 58051-900, Paraiba, Brazil; 4 Department of Mechanical Engineering, Federal University of Paraíba, João Pessoa 58051-900, Paraiba, Brazil; 5 Department of Production Engineering, Federal University of Campina Grande, Sumé 58540-000, Paraiba, Brazil; 6 Engineering Department, Rural Federal University of Semi-Arid (UFERSA), Caraúbas 59780-000, Rio Grande do Norte, Brazil; 7 Department of Natural Sciences, Mathematics and Statistics, Rural Federal University of Semi-Arid (UFERSA), Mossoró 59625-900, Rio Grande do Norte, Brazil; 8 Agroindustrial Management and Technology Department, Federal University of Paraíba, Bananeiras 58220-000, Paraiba, Brazil; 9 Department of Mechanical Engineering, Federal University of Campina Grande, Campina Grande 58429-900, Paraiba, Brazil

**Keywords:** biopolymer, curauá fiber, compatibility, biocomposites, morphology

## Abstract

For the purpose of renewable materials applications, Curauá fiber treated with 5% sodium hydroxide was added to high-density biopolyethylene, using an entirely Brazilian raw material of sugarcane ethanol. Polyethylene grafted with maleic anhydride was used as a compatibilizer. With the addition of curauá fiber, the crystallinity was reduced, possibly due to interactions in the crystalline matrix. A positive thermal resistance effect was observed for the maximum degradation temperatures of the biocomposites. When curauá fiber was added (5% by weight), the morphology showed interfacial adhesion, greater energy storage and damping capacity. Although curauá fiber additions did not affect the yield strength of high-density bio polyethylene, its fracture toughness improved. With the addition of curauá fiber (5% by weight), the fracture strain was greatly reduced to about 52%, the impact strength was also reduced, suggesting a reinforcing effect. Concomitantly, the modulus and the maximum bending stress, as well as the Shore D hardness of the curauá fiber biocomposites (at 3 and 5% by weight), were improved. Two important aspects of product viability were achieved. First, there was no change in processability and, second, with the addition of small amounts of curauá fiber, there was a gain in the specific properties of the biopolymer. The resulting synergies can help ensure more sustainable and environmentally friendly manufacturing of automotive products.

## 1. Introduction

The adverse environmental impacts caused by the massive use of non-biodegradable and non-renewable petroleum products has stimulated research into the use of biologically based resources. In this sense, we can cite bioplastics, for example. Bioplastics are an essential part of the bioeconomy and are an innovative and fast-growing industry that has the potential to decouple economic growth from resource depletion and environmental impact. The degradation of bioplastics in different environments has been described and reported as being mainly dependent on the composition of the bioplastics and environmental conditions. Incomplete degradation during waste management processes and leakage of bioplastics into the environment are becoming major concerns that need to be further investigated. The bioplastics industry is still very small in volume and relatively new compared to the regular plastics industry. Furthermore, regulations on bioplastics have been changing in recent years. Therefore, it is understandable that several present and future challenges related to the adoption of bioplastics can be identified.

Biopolymers and their technological processes have been extensively studied in several applications. Known primarily for their environmental benefits, biopolymers are considered promising alternatives to petroleum-based polymers, as most biopolymers help to reduce environmental pollution and CO_2_ gas emissions and thus minimize the effects of the greenhouse effect [1]. The study of natural biocomposites has opened up opportunities for the use of new high-performance raw materials. Materials such as green biocomposites (based on natural fibers and inorganic materials) can now replace conventional non-renewable materials based only on fossil sources such as oil and natural gas [2]. Most synthetic polymers are produced from non-renewable petrochemical sources, but concerns about the environment have made the use of polymers obtained from renewable sources more attractive. Biopolyethylene (B-HDPE), also known as “green polyethylene”, is obtained from ethylene generated by the fermentation and distillation of sugarcane juice, which initially produces ethanol. Ethanol is then dehydrated and transformed into ethylene [3]. “Green polyethylene” has a structure similar to polymers originating from the petrochemical route, but it is a technological innovation in the manufacture of plastic products, as green polyethylene absorbs CO_2_ from the atmosphere during the production cycle, in addition to reducing our dependence on raw materials of fossil origin [4].

In recent decades, an alternative chemistry has emerged, called “green chemistry”, which aims to reduce human impacts on the environment. Polymers are involved in this trend and various plastic materials from renewable sources are now used in a wide range of applications such as packaging, leisure, agriculture and biomedicine. Some of these polymers, biotechnological polymers such as polylactides (PLA), polyhydroxyalkanoates (PHA) and polysaccharides, combine renewability and biodegradability. However, although commercialization has started, for these biodegradable polymers to be truly competitive with conventional petroleum-based plastics, their mechanical and thermal properties and moisture sensitivity must to be improved [5]. The use of biopolymers produced from renewable raw material sources, such as corn, sugar cane, cellulose, chitin, among others, remains an alternative, and “green” polymers, such as B-HDPE, although not biodegradable, can be produced without impacting the balance of carbon dioxide (CO_2_) in nature. Carbon dioxide is captured by the sugarcane biomass from the atmosphere, and later, after the harvest, is released into the atmosphere through combustion, and is then captured again by the sugarcane biomass in the process of photosynthesis before the next harvest [6].

Despite all their advantages, biopolymers have certain technical limitations that hinder their processability and use as a final product. In general, these limitations are related to nature of the matrix and their interaction (adhesion) with reinforcement. Thus, many research groups are dedicated to studying the modification of biopolymers through the use of additives, which can enable their processing and use in various applications. The resulting composites are then studied to improve processability along with thermal resistance, mechanical strength and rheological parameters, compared to conventional polymers of petrochemical origin [7,8].

The replacement of synthetic fibers by natural fibers in composites is promising, natural fibers are non-toxic, renewable and generally inexpensive [9]. However, they also have certain disadvantages or limitations, such as remarkable variability in mechanical properties and low dimensional stability, the latter with high sensitivity to environmental effects, such as temperature and humidity variations, and lower processing temperatures. Brazil has great potential for the production of natural fibers, with a wide variety of lignocellulosic fibers, consisting of cellulose, hemicellulose (or polyoses), lignin, pectin, wax and water-soluble substances [10,11,12,13,14]. Cellulose, polyoses and lignin are generally responsible for the physical properties of a fiber, and also strongly influence the mechanical properties of the resulting biocomposite. Generally, biocomposites remain stable up to approximately 200 °C [15].

One of the main challenges facing polymer composites is achieving a satisfactory interfacial bonding with better mechanical properties. However, due to the hydrophilicity of natural fibers and the hydrophobicity of certain polymers used as matrices this can be difficult. The final properties of biocomposites depend mainly on the types of fibers used, their aspect ratios, volume fraction, orientation and adhesion strength at the fiber–matrix interface [16]. In order to improve polymer/fiber adhesion and reduce water absorption, the surface of the fibers can be modified using both physical and chemical methods [17,18]. The properties of these materials are generally improved by intensification of the interactions between the fiber and the matrix. The fiber/matrix interface plays an important role in the mechanical and physical properties of polymeric composites, since it is through the interface that the transfer of stresses from the matrix to the fibers occurs. The interaction between the components of a composite is a result of the characteristics of the adhesion interface. One of the effects that contribute to this adhesion is attraction between atoms with polar characteristics in both the matrix and the fiber reinforcement. Thus, to promote greater adhesion, the fibers are subjected to an appropriate surface pretreatment, and a compatibilizing agent is often added to the composite. Alkaline treatment is normally performed in an aqueous solution of NaOH [19].

The compatibilization agent acts at the polymer/fiber interface, promoting adhesion between the two phases. The most used compatibilizer for this purpose is polyethylene grafted with maleic anhydride (PE-g-MA) [20]. Like their isostructural analogues (maleic, fumaric, citraconic and itaconic acids) and amides, imides, esters and nitriles, these are polyfunctional monomers, used as grafts in copolymer synthesis. Various other thermoplastic polymers can also be used, such as polyolefins, polyamides and polysaccharides, as well as natural and synthetic rubbers. When producing engineering materials that require high performance, particularly in bioengineering and nanostructured materials, compatibilization is necessary [21].

It is well known that composites are used today for very diverse applications, such as biomedicine, civil construction and aerospace. Depending on the application, the matrix can be produced with metallic, ceramic materials. On the other hand, polymer composites reinforced with natural fibers are commonly used in varied applications such as automotive and aerospace, sports, medical implants and drug delivery, textiles, packaging, infrastructure and construction, upholstery and furniture, and many household products [22].

In the automotive industries, natural-fiber-reinforced biopolymer composites can offer several advantages over conventional composites, such as improved stability, reduced cost and weight, improved acoustic insulation and mechanical properties, reduced wear on machines and tools, less or no outgassing toxic/harmful materials, together with recyclability, renewability and eco-efficiency. Furthermore, biocomposites with natural fibers are widely accepted in transdermal drug delivery (TDD) applications due to their ease of addition into biological compounds, controlled drug release and permeation characteristics [23]. In biomedical and electronic applications, silk/chitosan composite films structured with nanofibers prepared by the easy solution casting technique exhibited better hydrogen bonding interactions, mechanical performance and biocompatibility with living cells. Films composed of biopolymers can potentially be used in biomedical and electronic screens due to their strong nanofibril structure [24]. Depending on the chemical composition, many other applications of biocomposites can be cited, for example, in the biosorption of organic matter and nutrients [25] and color removal from agro-industrial effluents [26].

Thus, based on the already mentioned information and the importance of studying biocomposites, the present work aimed to develop and evaluate biocomposites using a sustainable matrix of bio “green” high-density polyethylene (B-HDPE) and curauá fiber (CF), and to determine if low percentages of CF improve the properties of the biocomposite B- HDPE, with NaOH surface treatment and compatibility with PE-g-MA. The biocomposites were investigated for their structural (FTIR, XRD), thermal (DSC and TG), thermomechanical (DMA), morphological (SEM) and mechanical (tensile, bending, impact and hardness) properties.

## 2. Materials and Methods

### 2.1. Materials

High-density green polyethylene, trade name SGE7252 (B-HDPE), I’m Green *^®^*, was obtained from sugarcane ethanol and has the positive effect of reducing CO_2_ emissions into the atmosphere. It was provided by Braskem (Maceió, Brazil) and had a relative density of 0.952 g/cm^3^ and Fluidity Index, IF = 2.0 g/10 min^−1^ [27].

Natural fibers from the curauá (CF) plant were used as reinforcement. Curauá fibers (*Ananas erectifolius*) are composed of cellulose, hemicelluloses (polioses), lignin, pectins, waxes and water-soluble substances, and are found in abundance in the northern region of Brazil. The curauá fibers used had final lengths and diameters of 2.5 mm and 0.2 mm, respectively, and were generally purple in color.

### 2.2. Preparation of Curauá Fiber (CF)

**Figure 1:**
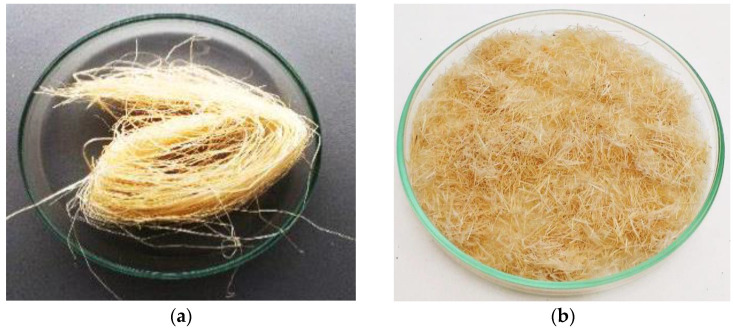
(**a**) Curauá long fibers before treatment and (**b**) fibers cut and treated with 5% NaOH.

Natural fibers from curauá (CF) plant were received in the form of long fibers, post-harvest and drying, without any previous treatment. Figure 1 shows the fibers (a) combed to remove husks and surface dirt and cut to 2.5 mm in length (b). An alkaline treatment was applied, a solution of sodium hydroxide (NaOH) at a concentration of 5% mass/volume. The fibers remained immersed in the NaOH solution, at a ratio of 10:1 (solution:fibers), for 2 h at a temperature of 50 °C. The fibers were washed with distilled water until the remaining washing solution had a pH of 7.0, then the fibers were dried in an oven at a temperature of 60 °C for 12 h according to [28].

### 2.3. Preparation of Biocomposites

The biocomposites were obtained from Green Polyethylene (B-HDPE) and curauá fibers (CF), treated with a 5% NaOH solution. In addition, 10% by weight of polyethylene grafted with maleic anhydride (PE-g-MA) was added to all biocomposites. Initially, the biocomposites were prepared by the melt-state method and processed in a modular, co-rotational extruder, model LSM 30.34 (LEISTRITZ Manufacturer, Cleveland, OH, USA), screw diameter 30 mm and L/D = 29. The process conditions were barrel temperature varying from 160 to 180 °C, screw speed of 150 RPM and feed rate of 2 kg/h. Then, the B-HDPE and the biocomposites were molded by injection in an ARBURG Injector—Goldem equipment (Lossburg, Germany), Tmold = 25 °C, Tzones = 180 to 200 °C, mold cooling time 5 s. The biocomposites were processed in the CF proportions of 1, 3 and 5% by mass. After this step, the samples were characterized. In Table 1 are cited the samples studied in this research.

### 2.4. Description

#### 2.4.1. Fourier Transform Infrared Spectroscopy (FTIR-ATR)

Lignocellulosic fibers have infrared transmittance bands characteristic of their constituents [29]. Thus, the curauá fiber was characterized by FTIR. The infrared spectra of the fibers were obtained in a BRUKER spectrophotometer, model FT-IR VERTEX 70 (Ettlingen, Germany), operating in the range of 4000 cm^−1^ and 500 cm^−1^.

#### 2.4.2. X-ray Diffraction (XRD)

The effects on the crystallinity of the B-HDPE/CF composites at different concentrations were evaluated using X-Ray diffraction (XRD). A RIGAKU X-ray diffractometer, MINI FLEX 2 (Wilmington, DE, USA) (Cu Kα radiation) operating in the range of 2Ɵ from 2 to 60 degrees, operating at 30 kv/15 mA, (0.02 min^−1^) was used.

#### 2.4.3. Thermal Analysis by Differential Scanning Calorimetry (DSC)

DSC analyses were carried out to identify the melting temperatures of the biocomposite and the degree of crystallinity. These were carried out in a SHIMADZU DSC-50 apparatus (Kyoto, Japan), heating the room temperature to 200 °C at a rate of 10 °C/min and under a nitrogen atmosphere (N_2_). To calculate the percentage of crystallinity, the ratio between the variations of the sample in the enthalpy of fusion (ΔHf_a_) and the enthalpy value of 100% for crystalline HDPE (ΔHf 100%) (293 J/g) [30,31] were calculated, as shown in Equation (1). In this calculation, it was assumed that HDPE and B-HDPE have an equivalent ΔHf_a_*,* since the difference between the two polymers consists only of the route used in the preparation of the monomer (ethylene).

(1) $$\left(\%\right)C=\left(\frac{\Delta {\mathrm{Hf}}_{a}}{\Delta {\mathrm{Hf}}_{100\%}}\right)\times 100,$$

#### 2.4.4. Thermogravimetry (TG and DTG)

The analyses were performed simultaneously in the TG/DTG SHIMADZU DTG 60H equipment (Kyoto, Japan), using about 5 ± 0.5 mg of sample, a heating rate of 10 °C/min, from room temperature to 800 °C and a gas flow of 50 mL/min, in a nitrogen atmosphere.

#### 2.4.5. Thermodynamic-Mechanical Analysis (DMA)

Dynamic thermomechanical tests of the BPEAD/PE-g-MA/CF biocomposites were carried out in DMA equipment, model Q800 - TA instruments (New Castle, USA), in bending mode, using oscillation amplitude of 20 μm, frequency of 1 Hz, heating rate of 2 °C/min, temperature range from 50 to 100 °C and specimens with dimensions of 57.5 × 12.73 × 3.12 mm.

#### 2.4.6. Analysis of Scanning Electron Microscopy (SEM)

The morphologies of treated and untreated fiber, B-HDPE and biocomposites were investigated using a SHIMADZU SSX-550 Superscan scanning electron microscope (Kyoto, Japan), with an accelerating voltage of 20 kV, in high vacuum. Fracture surfaces for B-HDPE and biocomposites were obtained after the IZOD impact test by being plated in gold for approximately 40 min.

#### 2.4.7. Mechanical Properties

Mechanical characterizations were performed using injected specimens. The tensile and bending tests were carried out in a SHIMADZU machine, MODEL AG-X (Kyoto, Japan), with a 10 kN load cell. The tensile test was performed at a speed of 50 mm/min in accordance with ASTM D638. For bending, the test speed was 2.0 mm/min per ASTM 790-96a. The IZOD impact-resistance test was carried out in accordance with ASTM D 256, in CEAST RESIL 5.5 equipment (Charlotte, NC, USA), with a 2.75 J pendulum, (the 2.5 mm notches were produced in a CEAST NOTSCHVIS slot machine). The Shore D hardness test was performed in accordance with ASTM D2240-15, with a fixed load of 50N. For each characterization, 5 specimens per sample were used.

## 3. Results and Discussion

### 3.1. FTIR

**Figure 2:**
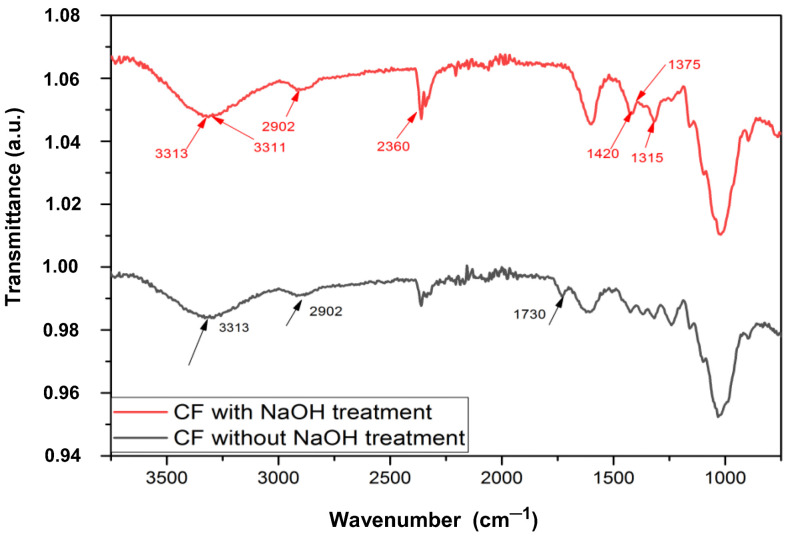
Curauá fiber FTIR (CF) with and without treatment with 5% NaOH.

Figure 2 presents the infrared spectra of curauá fibers with and without alkaline treatment (5% NaOH). The spectrum of the untreated fiber shows many absorption bands: a very weak and broad band at 3313 cm^−1^ typical of cellulose and water (OH) stretching, a very strong band at 2902 cm^−1^ typical of CH and others in the region of 1728 cm^−1^ typical of cellulose structural units [29]. In the fibers with alkaline treatment, the spectra show a decrease in intensity in the broad band at 3313–3311 cm^−1^, referring to the OH bonds of cellulose and water, as well as a reduction in the intensity of the bands around 2902 cm^−1^ and at 2360 cm^−1^ [32]. This result suggests a partial dissolution of the hemicellulose in the alkaline solution, corresponding, respectively, to the CH and C=O bonds of the ether group in the hemicellulose and in the lignin [5,29].

Partial removal of lignin is observed in treated fibers, indicated at 1417 cm^−1^, by the absence of the band, by the decrease in band intensity at 1315 cm^−1^ and by the absence of the band at 1375 cm^−1^, corresponding, respectively, to vibration of the benzene ring and stretching of the CH and CO bonds of the lignin acetyl group [5,33]. Previous studies using alkaline treatment on lignocellulosic fibers, such as curauá and sisal fibers, present similar characteristics, this is because more than 70% of the fiber structure is composed of cellulose [29]. FTIR results confirm that this type of treatment removes hemicellulose and lignin from fiber surfaces [34]. Chemical treatment or modification of major OH groups on the fiber surface leads to improvements in interaction with polymers in fiber-reinforced composites [35].

### 3.2. X-ray Diffraction (XRD)

**Figure 3:**
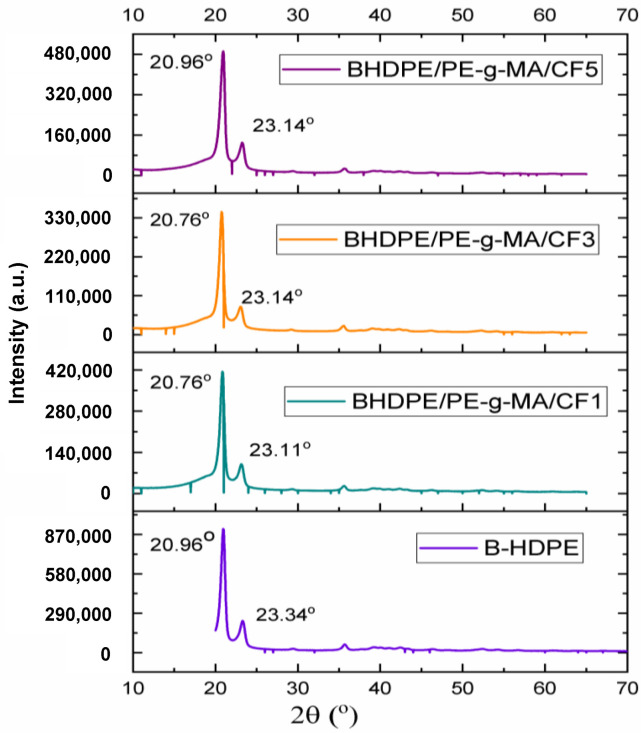
XRD patterns of B-HDPE and biocomposites.

The XRD analysis evaluates the changes that occur in the position, width and height of the peak, to infer changes in the crystalline structure (B-HDPE with CF incorporation). Figure 3 presents X-ray diffractograms in the high angle region for B-HDPE and biocomposites. The presence of crystalline peaks in region 2Ɵ around 20° and 23° [36] indicates respective diffractions in crystallographic planes (110) and (200), related to orthorhombic crystallization in high-density polyethylene. In general, the diffractograms revealed a decrease in peak intensity with the addition of CF, or a reduction in the crystallinity of B-HDPE. The characteristic peaks of the treatment with curauá fiber were also observed by [37] at 2Ɵ equal to 16°, 23° and 35°, and were attributed to the removal of amorphous materials from the fiber (lignin), promoted by the treatment with NaOH at 8% by weight. The XRD result was corroborated by the FTIR analysis (Figure 2) which showed that there was partial dissolution, and a small residual content of hemicellulose and lignin may have remained after the alkaline treatment with 5% NaOH by weight according to [32]. In the biocomposite diffractograms, it was not possible to observe the characteristic peak of the fiber at 2Ɵ = 16°; the peak at 23° is probably superimposed on the B-HDPE peak, and the peak at 2Ɵ = 35° has low intensity. Considering the half-height width of the peak at 2Ɵ = 20°, the peak widths of CF biocomposites (at 3 and 5% by weight) are even greater than B-HDPE. This may be related to the presence of fiber constituents (not removed with NaOH treatment), which reduce the regularity of chain arrangements in the polymeric matrix.

### 3.3. Thermal Analysis by Differential Scanning Calorimetry (DSC)

Table 2 presents the values of melting temperature I, melting enthalpy (ΔHf_a_) and degree of crystallinity (X_c_) for B-HDPE and biocomposites.

**Figure 4:**
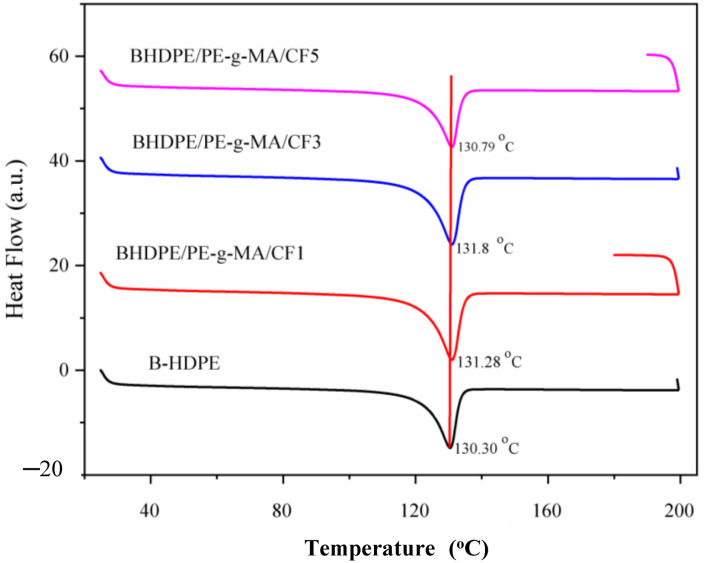
Melting thermograms of B-HDPE and biocomposites.

Figure 4 shows only a single endothermic peak for the matrix as well as for the biocomposites. The Tm value was practically unchanged with the addition of CF. The presence of a narrow peak is characteristic of semicrystalline materials such as B-HDPE [4]. The enthalpy of fusion decreased with the addition of CF, this reduction reveals that less heat is needed to melt the polymer. This reduces the energy required to process/produce the biocomposite and leads to cost savings [38]. The degree of crystallinity of B-HDPE decreased with CF additions, and crystallization may have been impaired by the presence of fiber. According to some authors [39,40], interactions are easier between the smaller chains of the polymer and the chemical components that are in the non-crystalline and more hydrophobic regions of the vegetable fiber, that is, in the low polarity regions of the lignin macromolecule. As observed in the FTIR analysis, partial dissolution of the CF amorphous phase probably occurred. Thus, interactions between CF and amorphous regions of B-HDPE probably contributed to the decrease in crystallinity of the biocomposite. This result was corroborated by the XRD analysis, which revealed a reduction in the crystallinity of B-HDPE with the addition of CF.

### 3.4. Thermogravimetry

**Figure 5:**
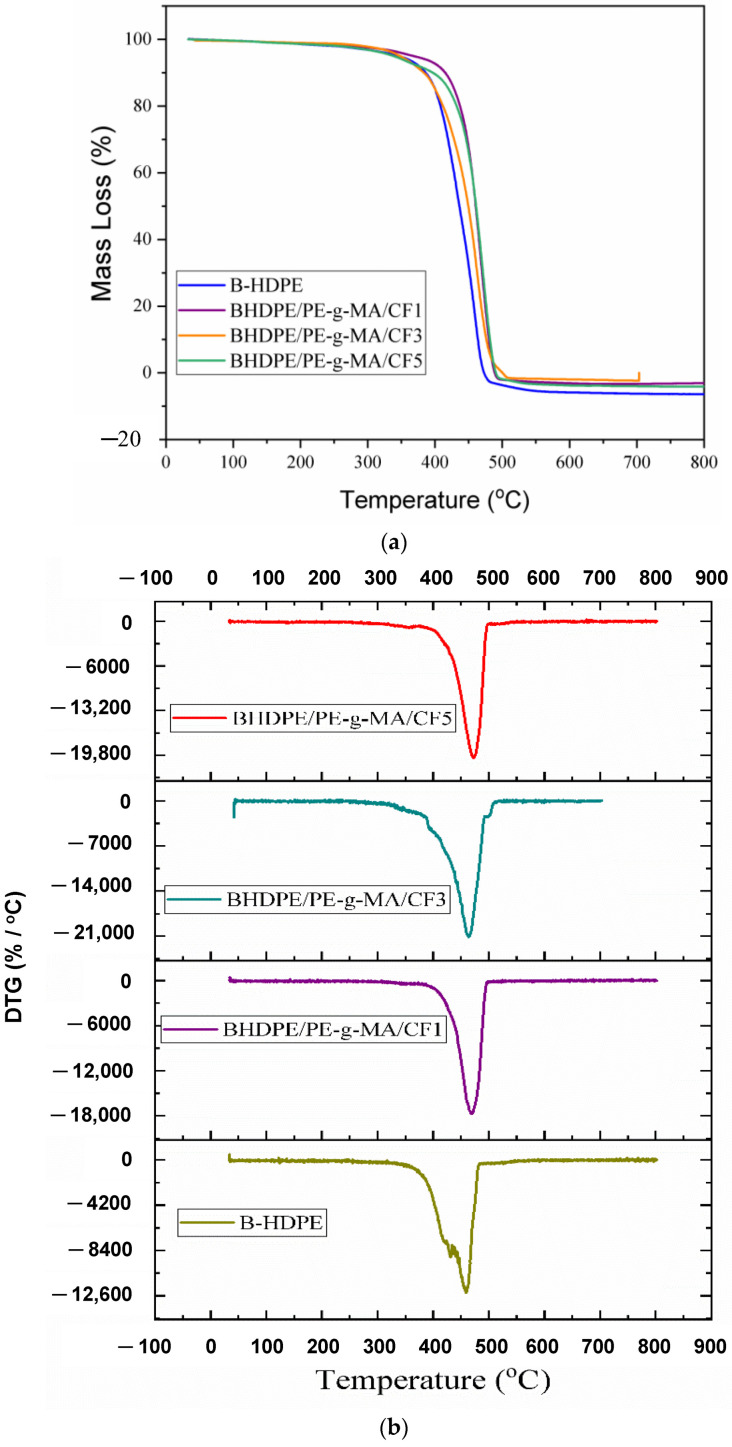
(**a**) TGA and (**b**) DTG curves of B-HDPE and biocomposites.

Figure 5 shows the TG and DTG curves of the samples. B-HDPE and biocomposites have only one degradation step, characteristic of the polymer. The first mass loss due to B-HDPE degradation began (T_onset_) at approximately 391.64 °C and increased with CF additions, with a considerable improvement of 22.31% for the B-HDPE/PE-g-MA/CF5 biocomposite. This result suggests that the processing of these biocomposites can occur without risk of thermal degradation at temperatures normally used in polymer processing equipment around 200 °C. The same trend of temperature increase in the biocomposites was observed at 80 and 50% of mass loss, T(80) and T(50), as the final degradation temperature (T_final_) increased. Although the increases show little difference between the biocomposites, the CF 3% by mass showed the lowest thermal resistance, see Table 3. Regarding the maximum rate of mass loss as a function of temperature, it was observed that the maximum degradation temperature of the B-HDPE (T_max_) increased with additions of CF, mainly with the addition of 5%, which showed an increase of 13 °C. On the other hand, high-density biopolyethylene (BHDPE)/liquid hydroxylated polybutadiene (LHPB)-based curauá fiber biocomposites have lower maximum degradation temperatures than the polymeric matrix [40]. Likewise, HDPE/curauá biofiber biocomposites, plasticized with castor and canola oil, showed a reduced maximum rate of mass loss in relation to the polymeric matrix [41]. Based on [40,41], the modifications made to the polymeric matrix by the curauá fiber reduced the thermal resistance of the biocomposites. When using plant fiber without surface treatment [42], no synergistic effect on the thermal stability of HDPE/plant fiber composites due to the presence of amorphous fiber constituents has been reported. In the present work, very low percentages of CF were used. However, surface treatment of CF with NaOH partially removed amorphous constituents such as lignin (see FTIR analysis). In addition, the compatibility with PE-g-MA promoted interfacial adhesion with a consequential increase in resistance to thermal degradation in the biocomposites in relation to B-HDPE, which occurred throughout the evaluated temperature range.

### 3.5. Analysis by Scanning Electron Microscopy (SEM)

**Figure 6:**
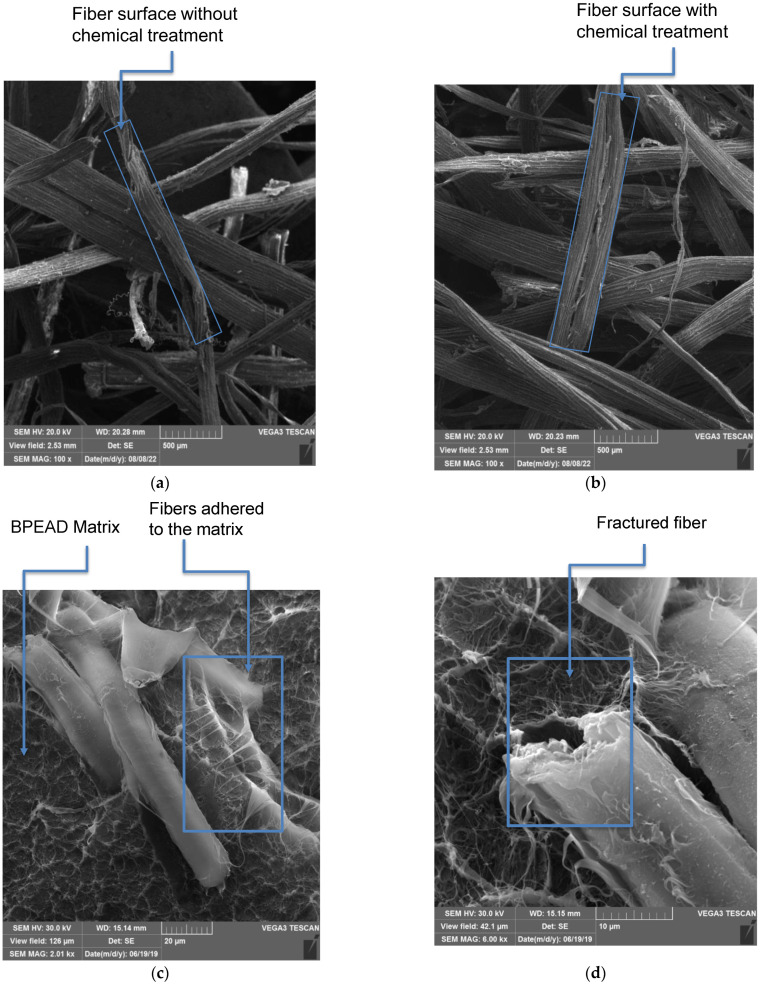
(**a**) SEM micrographs of the curauá fibers without alkaline treatment, (**b**) with alkaline treatment and (**c**,**d**) B-HDPE/PE-g-MA/CF1 biocomposites.

**Figure 7:**
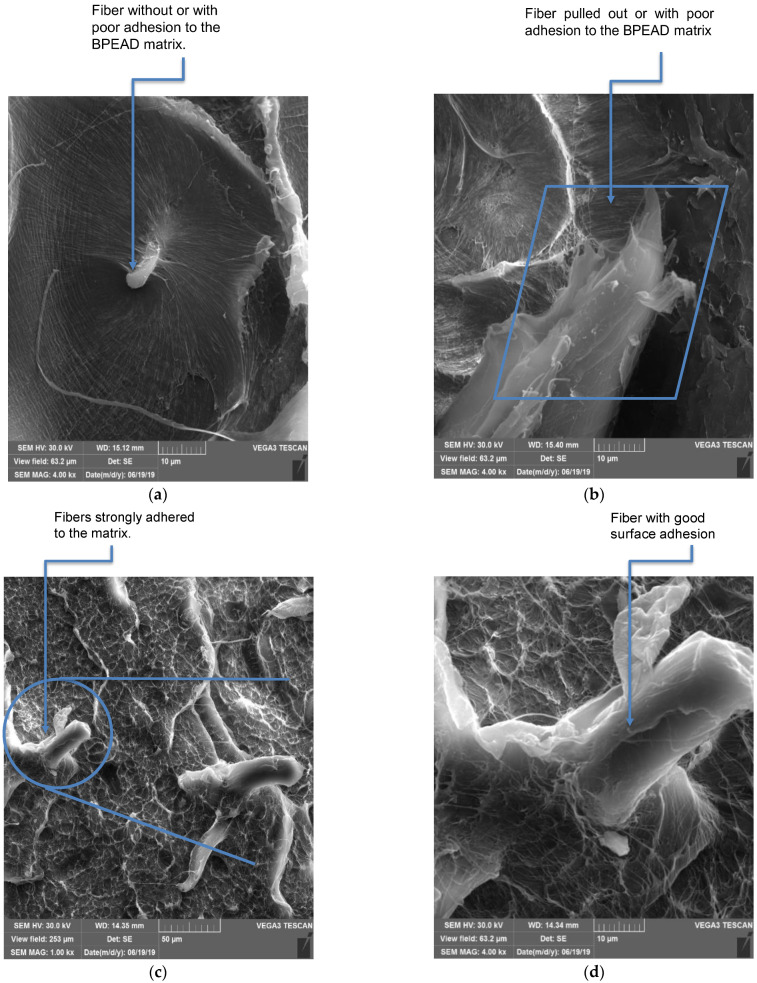
SEM micrographs of biocomposites: (**a**,**b**) B-HDPE/PE-g-MA/CF3 and (**c**,**d**) B-HDPE/PE-g-MA/CF5.

The surface of the curauá fiber was scanned by SEM, both without (Figure 6a) and with (Figure 6b) alkaline treatment. When comparing the CF images, it is clear that the alkaline treatment increased the surface roughness, leaving it more exposed. This suggests the increased potential for NaOH-treated CF to interact with the polymer. After the impact test, the surfaces of the biocomposites were also analyzed by SEM to correlate the results after the impact with the fracture surfaces shown in Figure 6 and Figure 7. In all micrographs there is evidence that the CF fibrils were separated from the beam of microfibers [43]. This was also reported by [44] in polypropylene/vegetable fiber composites compatibilized with PE-g-MA. In the micrographs of the B-HDPE/PE-g-MA/CF1 biocomposite, detachment of the fibers, fibers adhered to the matrix (Figure 6c) and fractured fibers (Figure 6d) were observed. The micrographs of Figure 7a,b indicate that the 3% CF biocomposite showed both fiber–matrix adhesion (Figure 7a) and relatively loose fiber, as can be seen in the detail of Figure 7b. Fiber detachment and pullout mechanisms are predominant during impact energy absorption, and greater lengths of pulled fibers contribute to higher impact strength of the composite [45]. The B-HDPE/PE-g-MA/CF5 biocomposite showed pulled fibers (Figure 7c) and fibers adhered to the matrix (Figure 7d). This composition showed greater interfacial adhesion than the other biocomposites. The CF–matrix interaction is chemical, and the chemical bond promoted adhesion of the matrix to the fiber surfaces even after being pulled out (Figure 7c,d). The justification for this is related both to the exposure of hydroxyl groups on the surface of the fiber (due to the chemical treatment with NaOH) and to the PE-g-MA compatibilizer. Thus, a part of the compatibilizer remained chemically bound to the hydroxyls on the fiber surface, and the other part of the compatibilizer remained molecularly interacting with the matrix, leading to interfacial strengthening as detailed by [46]. This adhesion mechanism was also observed with the addition of a PE-g-MA compatibilizer to a polyethylene compatibilizer with Al_2_O_3_ inorganic fiber, resulting in an improvement in interfacial adhesion through the polar interactions of the compatibilizer’s acrylic acid with the group’s surface fiber hydroxyl [47]. With no interfacial adhesion between the matrix and plant fiber, long pull lengths (with voids) were observed due to fiber removal at fracture, the pulled fiber surfaces remained clean. These aspects indicate poor interfacial adhesion; see Sarkhel and Choudhury [48]. In the present work, interfacial adhesion was observed in the biocomposites (notably in B-HDPE/PE-g-MA/CF5) and the stress transfer between the polymer and the fibers was probably more effective in this biocomposite.

### 3.6. Dynamic Mechanical Analysis (DMA)

**Figure 8:**
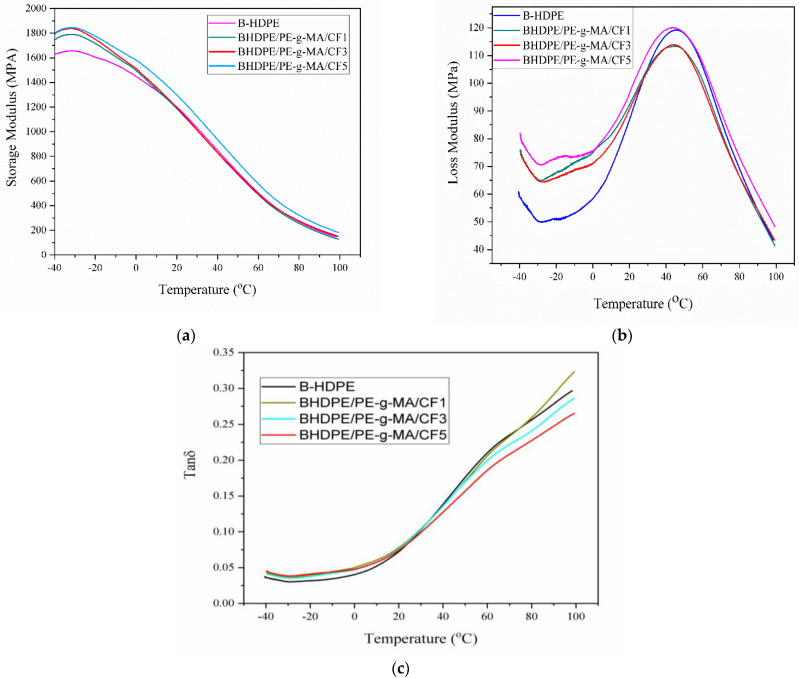
Typical DMA analysis curves: (**a**) storage modulus, (**b**) loss modulus and (**c**) tan δ.

In polymer composites, the dynamic mechanical properties (storage modulus, loss modulus and tan Ω) are sensitive to the amount of fiber, the presence of additives (compatibilizers), fiber orientation and interfacial alteration due to adhesion [48,49]. Figure 8 shows the dynamic mechanical properties of B-HDPE and biocomposites.

The storage modulus (E′) is related to the resistance capacity of the material to loading. The addition of CF increased the E′ of the B-HDPE, and in the temperature range from −40 to −27 °C, biocomposites 3 and 5% showed an increase in the E′ of 12% in relation to the pure polymer. In the entire temperature range, BPEAD/PE-g-MA/CF5 showed E′ greater than B-HDPE and the other biocomposites. The energy dissipated in the form of heat during deformation represents the loss modulus (E″) and is very sensitive to the molecular architecture of the polymer. HDPE has three characteristic relaxations at −110 °C (γ), −30 °C (β) and the third named (α) involves a wider temperature range, from 50 to 120 °C.

Relaxation (α) is associated with the mobility of long chain segments in the crystalline phase [50,51], being more susceptible than the other transitions due to the restrictions imposed on the segmental mobility of the polymer with the addition of reinforcement [52]. By the E″ curves, both the B-HDPE and the biocomposites present relaxation (α) around 44 °C, agreeing with [36] who reported relaxation around 35 °C. The addition of 5% CF increased the peak height of the loss modulus in relation to the other biocomposites, indicating less flexibility due to the greater restriction of segmental mobility in the polymeric matrix due to the presence of the fiber [53]. Furthermore, the increase in E″ height is indicative of interfacial interaction [54]. Of course, consistent with the interfacial adhesion observed in Figure 7d, this effect also occurred (with the addition of 5% CF) in B-HDPE. The addition of CF amplified the E″ peak in relation to B-HDPE, and according to [43], this is due to the lower viscous dissipation of the polymer in the presence of the fiber. Tan δ is the ratio between E″/E′, and represents the damping property of the material; if its value is small, this suggests resistance in the interfacial connection between the matrix and the fiber [54]. Compositions with 3% and 5% CF showed lower values of tan δ than B-HDPE, suggesting a greater damping capacity in these compositions to reduce the effects of vibration, noise, etc. [55]. This reduction was observed for BPEAD/PE-g-MA/CF5, as in other composites based on plant fibers, and is a compatibilization effect [41,48].

The DMA results correlated with those obtained in thermogravimetry, as the greater thermal resistance of the biocomposites is probably related to the interfacial interaction. B-HDPE/PE-g-MA/CF1 was the least interesting for applications that require the ability to reduce/dissipate vibrational energy, and presented a damping behavior similar to that of the matrix.

### 3.7. Mechanical Properties

**Figure 9:**
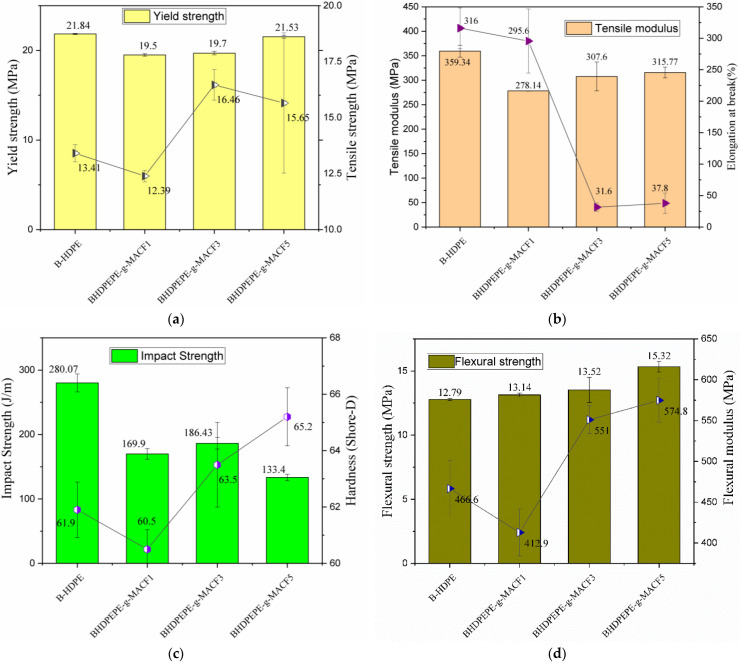
Mechanical properties of B-HDPE and biocomposites: (**a**) yield strength and rupture stress; (**b**) modulus under tension and deformation until failure; (**c**) impact strength and hardness; (**d**) flexural strength and flexural modulus.

Figure 9 shows graphs comparing the mechanical properties obtained for traction, flexion, impact resistance and hardness of B-HDPE and biocomposites.

For better understanding, flexural strength is a material property that can be defined as the stress just before it yields in a flexure test; flexural modulus represents the tendency for a material to resist bending; tensile strength corresponds to the maximum stress that a material can withstand while being axially deformed before rupture; tensile modulus corresponds to the mechanical property that is related to the stiffness (tensile or compressive) of the solid material.

The yield strength values of the biocomposites remained virtually constant, with very little variation and a slight improvement for the 5 wt% CF biocomposite. The tensile strengths at break for the biocomposites with 3 and 5 wt% CF were 16.46 and 15.65 MPa, respectively, compared to the B-HDPE of 13.41 MPa, with respective improvements of 22.7 and 16.7%. The elongation at break (Figure 9b) was drastically reduced for the composites of 3 and 5% by weight of CF, suggesting the reinforcing effect of the curauá fiber even at low concentrations. On the other hand, this result indicates that increasing the fiber concentration reduces the tenacity of the matrix. The difficulty with CF-supplemented biocomposites (3% and 5% by weight) deforming to fracture is probably related to the interaction with the PE-g-MA which acted as a matrix-reinforcing bridge. The effect of compatibilization on reducing stress to fracture was also observed in biocomposites of B-HDPE with thyme herbs compatibilized with poly(ethylene-co-glycidyl methacrylate-PE-g-MA). For example, with the addition of a 10% fiber reinforcement, fracture strain was drastically reduced in composites (from 520% (bio HDPE) to 8.47%) [27]. It is noteworthy that the types and contents of fibers and the processes for obtaining the biocomposites were different. This can have significant effects on the strength of green polyethylenes. With the addition of 1% CF, the modulus of elasticity under tension (Figure 9b) was reduced by about 22%, and smaller reductions of 14 and 12% were observed, respectively, for CF concentrations of 3 and 5% by weight. Beltrami and others [28] obtained an increase in the modulus of elasticity under tension of around 30% for poly(3-hydroxybutyrate) (PHB) with the incorporation of 20% by weight of CF treated with 5% NaOH. It can be inferred that the modulus of elasticity is greatly influenced by the fiber content. For B-HDPE compatible with PE-g-MA, higher CF contents in the biocomposites showed better reinforcement potential.

The impact strength of fiber-reinforced composites is sensitive to the constituents themselves, interfacial interaction, composite production processes and even test conditions [56]. The interface is an important factor in the impact strength of the composite, which is reduced with interfacial adhesion [44]. Composites of PLA (polylactic acid) with plant fibers investigated by [47] revealed that fiber pullout occurred due to a weak interface, leading to an increase in impact resistance. Compared to fiber fracture, these energy absorption mechanisms increase the toughness of composites [51,57]. The effect of compatibilizers on the impact strength of high-density polyethylene composites based on carbon fiber was investigated by [57]. Due to the pulling out of the fibers in the composites, the highest energy absorption was observed in those with the lowest proportion of compatibilizer. Figure 9c presents the results of the impact resistance, in which the B-HDPE presents a value of 280.07 J/m, (when it was investigated by [41], it presented a value of 234 J/m, a gain of 19.7%). This difference can be explained by the types of processes used to obtain the composites. In the present study, it is possible that the molecular orientations that occurred during injection molding acted as a barrier to crack propagation, initiated from the notch located perpendicularly in the sample. The B-HDPE/PE-g-MA/CF1 biocomposite showed the highest impact resistance of the biocomposites, and its fracture surface revealed the presence of impact energy absorption mechanisms, such as fiber detachment and fracture. The addition of 5% CF led to a more pronounced reduction of around 52% in relation to the pure polymer, decreasing its deformation capacity according to the reduction in deformation until fracture. As observed in the SEM, the short-pulled fibers were well adhered to the matrix, possibly hindering the occurrence of more significant tenacity mechanisms. This result is in agreement with that reported by [43], and suggests that the compatibilization hinders the detachment of the fiber from the fiber–matrix interface. Finally, in the present work, the dynamic-mechanical analysis also indicated less flexibility, suggesting (in this composition) the effects of reinforcement.

Shore D hardness evaluates the puncture resistance of the surface. Figure 9c reveals a hardness for B-HDPE around 62. Biocomposites with 1 and 3% fiber showed values very close to pure polymer, while B-HDPE/PE-g-MA/CF5 showed an increase of 5.3% in relation to the parent company. A good dispersion of the reinforcement within the matrix, with greater interfacial adhesion and consequently a decrease in the amount of voids, leads to an increase in the hardness of the composite [58]. The Shore D hardness of the biocomposites was slightly higher than that of B-HDPE. The performance of 5% by weight CF in polymer reinforcement is confirmed by its decreasing impact strength and increasing hardness. In addition, due to the morphologies of the B-HDPE/PE-g-MA/CF5 fracture surfaces (Figure 7c,d), the interfacial interaction appeared stronger than in the other biocomposites, justifying the improvements observed in relation to the pure polymer.

Figure 9d presents the bending results, the addition of CF led to a small increase in maximum stress around 5% for the biocomposites with 1 and 3% CF by mass, and the B-HDPE/PE-g-MA/CF5 showed an increase of 22.13% compared to B-HDPE. Addition of 3% CF by mass resulted in an increase in modulus of 18.10%. The BPEAD/PE-g-MA/CF5 biocomposite showed a more positive effect, with an increase of 23.19%. From CF 3% by weight, the reinforcing capacity was also observed during bending stress. The mechanical response during bending of the biocomposites was superior to the tensile strength. These results are in agreement with [27,57], who suggested that in particulate composites, the stress during flexion is less aggressive than under tension and involves increases in both flexural modulus and strength.

## 4. Conclusions

With growing environmental awareness, studies focused on eco-friendly materials (renewable, recyclable and sustainable) play an important role. This highlights the emergence of a new type of material, with the replacement of non-renewable materials, which are more difficult to degrade or are non-degradable, with renewable and sustainable materials. The development of such materials is not only a great motivating factor for research development, but also to meet global concepts of sustainable development.

The present work focuses on the study of biocomposite reinforced by vegetable fiber. Herein, small amounts of short curauá fiber were used with the compatibilizing agent PE-g-MA to modify the properties of B-HDPE. According to the FTIR results, modifying the fiber surface with NaOH led to partial dissolution of hemicellulose and lignin. XRD results detected fiber peaks with NaOH treatment. The presence of fiber reduced the degree of crystallinity of the polymer and, as suggested by XRD, a decrease of about 18% was observed in the DSC analysis. The biocomposites were more resistant to temperature than the pure polymer, bringing an increase of 13 °C to the maximum degradation temperature with the addition of 5% by weight of fiber. After the impact test, the fracture surface (assessed by SEM) revealed that the addition of 1% by weight of fiber promoted detachment and fiber fracture mechanisms, while the addition of 5% by weight led to greater interfacial adhesion. DMA analysis suggested interfacial interaction between B-HDPE and CF due to an increase in E’ and a decrease in tan Ω, mainly for the biocomposite with 5% CF by weight. The mechanical properties under tension indicated that the yield strength of B-HDPE remained essentially unchanged, while the tensile strength values of the biocomposites with 3 and 5% by weight of CF (at failure), respectively, increased by about 22.7 and 15.65%. The elongation at break was drastically reduced as a result of compatibilization. The elastic modulus of B-HDPE was reduced, although the 5 wt% CF biocomposite was less affected. The impact resistance decreased concomitantly with the increase in hardness. For bending, the results demonstrated the performance of curauá fiber as a reinforcement (3 and 5% by weight) for B-HDPE, with increases in both maximum tension and modulus. We conclude that the moderate use of curauá fiber allowed the use of biocomposite processing parameters equivalent to those used for pure polymers, without altering the processability of B-HDPE. The value of this work lies in demonstrating that small amounts of curauá fiber can have synergistic effects by increasing the properties of the matrix, such as resistance to thermal degradation, hardness, mechanical properties under bending and damping characteristics by DMA. All these features are of interest for biocomposite applications in the automotive sector.

## Figures and Tables

**Table 1:** Sample labeling.

Designation	FC (%)	PE-g-MA (%)
B-HDPE	0	0
B-HDPE/PE-g-MA/CF1	1	10
B-HDPE/PE-g-MA/CF3	3	10
B-HDPE/PE-g-MA/CF5	5	10

**Table 2:** DSC results of B-HDPE and biocomposites.

Samples	Tm (°C)	ΔHf_a_ (J/kg)	X_c_ (%)
B-HDPE	130.30	120.60	41.16
B-HDPE/PE-g-MA/CF1	131.28	100.09	34.16
B-HDPE/PE-g-MA/CF3	131.80	99.07	33.81
B-HDPE/PE-g-MA/CF5	130.79	98.50	33.61

**Table 3:** T_onset_, temperatures at 80% and 50% mass loss, T_max_ and T_endset_, of TG and DTG of B-HDPE and its biocomposites.

	B-HDPE	B-HDPE/PE-g-MA/CF1	B-HDPE/PE-g-MA/CF3	B-HDPE/PE-g-MA/CF5
T_onset_ (°C)	391.64	419.36	405.81	413.95
T(80) (°C)	408.21	437.77	411.84	432.02
T(50) (°C)	436.18	460.23	449.37	460.98
T_max_ (°C)	459.87	469.18	463.95	472.86
T_endset_ (°C)	483.91	491.27	490.31	490.31

## Data Availability

Data supporting the findings of this study are available upon request from the authors.

## References

[1] Vargas Mores G., Pauletto C., Finocchio C.P.S., Barichello R., Pedrozo E.A. (2018). Sustainability and innovation in the Brazilian supply chain of green plastic. J. Clean. Prod..

[2] Raquez J.M., Habibi Y., Murariu M., Dubois P. (2013). Polylactide (PLA) based nanocomposites. Prog. Polym. Sci..

[3] Zambanini M.E., Bresciani L.P., Silva Pereira R., de Souza D.A., Ortega G. (2014). Sustainability and innovation: A study on green plastic. Rev. Agribus. Environ..

[4] Hassan M.L., Rowell R.M., Fadl N.A., Yacoub S.F., Christainsen A.W. (2000). Preparation and characterization of esterified bagasse fibers. J. Appl. Polym. Sci..

[5] Delgado J.M.P.Q., Lima A.G.B., Carvalho L.H. (2022). Moisture Transport in Polymer Composites Materials: Computactional Modeling and Experiments.

[6] Brito G.F., Agrawal P., Araújo E.M., Mélo T.J.A. (2011). Biopolymers, biodegradable polymers and green polymers. Rev. Eletrôn. Mater. Process..

[7] Wong S., Shanks R., Hodzic A. (2004). Interfacial improvements in poly (3 hydroxybutyrate)-flax fiber composites with hydrogen bonding additives. Compos. Sci. Technol..

[8] Chen L.J., Wang M. (2002). Production and evaluation of biodegradable composites based on PHB–PHV copolymer. Biomaterials.

[9] Monteiro S.N., Aquino R.C.M.P., Lopes F.P.D. (2008). Performance of curaua fibers in pullout tests. J. Mater. Sci..

[10] Rahman M.R., Hamdan S., Jayamani E., Kakar A., Bakri M.K.B., Yusof F.A.B.M. (2019). Tert-butyl catechol/alkaline-treated kenaf/jute polyethylene hybrid composites: Impact on physico-mechanical, thermal and morphological properties. Polym. Bull..

[11] Cruz J., Fangueiro R. (2016). Surface modification of natural fibers: A review. Procedia Eng..

[12] Satyanarayana K.G., Guimarães J.L., Wypych F. (2007). Studies on lignocellulosic fibers of Brazil. Part I: Source, production, morphology, properties and applications. Compos. Part A Appl. Sci. Manuf..

[13] Salazar V.L.P., Leão A.L., Rosa D.S., Gomez J.G.C., Alli R.C.P. (2011). Biodegradation of Coir and Sisal Applied in the Automotive Industry. J. Polym. Environ..

[14] Megiatto J., Jackson D., Hoareau W., Gardrat C., Frollini E., Castellan A. (2007). Sisal fibers: Surface chemical modification using reagent obtained from a renewable source; characterization of hemicellulose and lignin as model study. J. Agric. Food Chem..

[15] Satyanarayana K.G., Arizaga G.G.C., Wypych F. (2009). Biodegradable composites based on lignocellulosic fibers-An overview. Prog. Polym. Sci..

[16] Terzopoulou Z.N., Papageorgiou G.Z., Papadopoulou E., Athanassiadou E., Alexopoulou E., Bikiaris D.N. (2015). Green composites prepared from aliphatic polyesters and bast fibers. Ind. Crop. Prod..

[17] Sever K., Erden S., Gülec H.A., Seki Y., Sarikanat M. (2011). Oxygen plasma treatments of jute fibers in improving the mechanical properties of jute/HDPE composites. Mater. Chem. Phys..

[18] Poletto M., Ornaghi H.L., Zattera A.J. (2015). Thermal Decomposition of Natural Fibers: Kinetics and Degradation Mechanisms. React. Mech. Therm. Anal. Adv. Mater..

[19] Bledzki A.K., Gassan J. (1999). Composites reinforced with cellulose based fibres. Prog. Polym. Sci..

[20] da Silva F.S., da Silva W.A., Luna C.B.B., Ferreira E.S.B., Araújo E.M. (2021). Production of biopolyethylene/wood flour biocomposites compatible with PE-g-MA. Evaluation of maleic anhydride content in mechanical and thermomechanical properties. Res. Social Dev..

[21] Rzayev Z.M.O. (2011). Graft copolymers of maleic anhydride and its isostructural analogues: High performance engineering materials. arXiv.

[22] Huang H., Dean D. (2019). 3-D Printed Porous Cellulose acetate Tissue Scaffolds for Additive Manufacturing. Addit. Manuf..

[23] Syracuse V., Blanco I. (2020). Bio-Polyethylene (Bio-PE), Bio-Polypropylene (Bio-PP) and Bio-Poly (ethylene terephthalate)(Bio-PET): Recent developments in bio-based polymers analogous to petroleum-derived ones for packaging and engineering applications. Polymers.

[24] Mota J.C., Almeida M.A., Alencar V.C., Curi W.F. (2009). Environmental, economic and social impacts and benefits of biofuels: A global view. Eng. Ambient. Espírito St. Pinhal.

[25] Jagaba A.H., Kutty S.R.M., Baloo L., Hayder G., Birniwa A.H., Taha A.T.B., Mnzool M., Lawal I.M. (2022). Waste Derived Biocomposite for Simultaneous Biosorption of Organic Matter and Nutrients from Green Straw Biorefinery Effluent in Continuous Mode Activated Sludge Systems. Processes.

[26] Jagaba A.H., Kutty S.R.M., Abubakar S., Birniwa A.H., Lawal I.M., Umaru I., Usman A.K., Yaro N.S.A., Al-Zaqri N., Al-Maswari B.M. (2022). Synthesis, Characterization, and Performance Evaluation of Hybrid Waste Sludge Biochar for COD and Color Removal from Agro-Industrial Effluent. Separations.

[27] Montanes N., Garcia-Sanoguera D., Segui V.J., Fenollar O., Boronat T. (2018). Processing and characterization of environmentally friendly composites from biobased polyethylene and natural fillers from thyme herbs. J. Polym. Environ..

[28] Beltrami L.V.R., Cristine Scienza L., Zattera A.J. (2014). Effect of alkaline treatment of Curauá fibers on the properties of biodegradable matrix composites. Polímeros.

[29] Tomczak F., Satyanarayana K.G., Sydenstricker T.H.D. (2007). Studies on lignocellulosic fibers of Brazil: Part III–Morphology and properties of Brazilian curauá fibers. Compos. Part A Appl. Sci. Manuf..

[30] Marcovich N.E., Villar M.A. (2003). Thermal and mechanical characterization of linear low-density polyethylene/wood flour composite. J. Appl. Polym. Sci..

[31] Perez I.S.B., Manrich S. (2008). Effect of adding different copolymers to post-consumer HDPE/HIPS blends: Phase morphology and thermal properties. Polymers.

[32] Ibrahim N.A., Hadithon K.A., Abdan K. (2010). Effect of fiber treatment on mechanical properties of kenaf fiber-ecoflex composites. J. Reinf. Plast. Compos..

[33] Hossain M.K., Dewan M.W., Hosur M., Jeelani S. (2011). Mechanical performances of surface modified jute fiber reinforced biopol nanophased green composites. Compos. Part B Eng..

[34] Hoareau W., Trindade W.G., Siegmund B., Castellan A., Frollini E. (2004). Sugar cane bagasse and curaua lignins oxidized by chlorine dioxide and reacted with furfuryl alcohol: Characterization and stability. Polym. Degrad. Stab..

[35] Lahor A., Nithitanakul M., Grady G.P. (2004). Blends of low-density polyethylene with nylon compatibilized with a sodium-neutralized carboxylate ionomer. Eur. Polym..

[36] Gutiérrez M.C., De Paoli M.A., Felisberti M.I. (2012). Biocomposites based on cellulose acetate and short curauá fibers: Effect of plasticizers and chemical treatments of the fibers. Compos. Part A Appl. Sci. Manuf..

[37] Boronat T., Fombuena V., Garcia-Sanoguera D., Sanchez-Nacher L., Balart R. (2015). Development of a biocomposite based on green polyethylene biopolymer and eggshell. Mater. Des..

[38] Castro D.O., Frollini E., Marini J., Ruvolo-Filho A. (2013). Preparation and characterization of biocomposites based on curauá fiber, high-density biopolyethylene (BPEAD) and hydroxylated liquid polybutadiene (PBHL). Polymers.

[39] Moly K.A., Radusch H.J., Androsh R., Bhagawan S.S., Thomas S. (2005). Nonisothermal crystallisation, melting behavior and wide angle X-ray scattering investigations on linear low density polyethylene (LLDPE)/ ethylene vinyl acetate (EVA) blends: Effects of compatibilization and dynamic crosslinking. Eur. Polym. J..

[40] Castro D.O., Ruvolo-Filho A., Frollini E. (2004). Materials prepared from biopolyethylene and curaua fibers: Composites from biomass. Polym. Test..

[41] Castro D.O., Passador F., Ruvolo-Filho A., Frollini E. (2017). Use of castor and canola oils in biopolyethylene curauá fiber. Compos. Part A Appl. Sci..

[42] Barman A., Shrivastava N.K., Khatua B.B., Ray B.C. (2015). Green composites based on high-density polyethylene and S accharum spontaneum: Effect of filler content on morphology, thermal, and mechanical properties. Polym. Compos..

[43] Bos H.L., Müssig J., van den Oever M. (2006). Mechanical properties of short-flax-fibre reinforced compounds. Compos. Part A Appl. Sci..

[44] Thomason J.L., Vlug M.A. (1997). Influence of fiber length and concentration on the properties of glass fibre-reinforced polypropylene: 4. Impact properties. Compos. Part A Appl. Sci..

[45] Robledo-Ortíz J.R., González-López M.E., Rodrigue D., Gutiérrez-Ruiz J.F., Prezas-Lara F., Pérez-Fonseca A.A. (2020). Improving the Compatibility and Mechanical Properties of Natural Fibers/Green Polyethylene Biocomposites Produced by Rotational Molding. J. Polym. Environ..

[46] Zhang S., Ke Y., Cao X., Ma Y., Wang F. (2012). Effect of Al_2_O_3_ fibers on the thermal conductivity and mechanical properties of high density polyethylene with the absence and presence of compatibilizer. J. Appl. Polym. Sci..

[47] Bax B., Müssig J. (2008). Impact and tensile properties of PLA/Cordenka and PLA/flax composites. Compos. Sci. Technol..

[48] Sarkhel G., Choudhury A. (2008). Dynamic mechanical and thermal properties of PE-EPDM based jute fiber composites. J. Appl. Polym. Sci..

[49] Faulstich de Paiva J.M., Frollini E. (2006). Unmodified and modified surface sisal fibers as reinforcement of phenolic and lignophenolic matrices composites: Thermal analysis of fibers and composites. Macromol. Mater. Eng..

[50] Karger-Kocsis J., Mahmood H., Pegoretti A. (2015). Recent advances in fiber/matrix interphase engineering for polymer composites. Prog. Mater. Sci..

[51] Savas L.A., Tayfun U., Dogan M. (2016). The use of polyethylene copolymers as compatibilizers in carbon fiber reinforced high density polyethylene composites. Compos. Part B Eng..

[52] López-Manchado M.A., Biagitti J., Kenny J.M. (2002). Comparative study of the effects of different fibers on the processing and properties of ternary composites based on PP-EPDM blends. Polym. Compos..

[53] Palanivel A., Veerabathiran A., Duruvasalu R., Iyyanar S., Velumayil R. (2017). Dynamic mechanical analysis and crystalline analysis of hemp fiber reinforced cellulose filled epoxy composite. Polímeros.

[54] de Oliveira Santos R.P., Castro D.O., Ruvolo-Filho A.C., Frollini E. (2014). Processing and thermal properties of composites based on recycled PET, sisal fibers, and renewable plasticizers. J. Appl. Polym. Sci..

[55] Wang M., Joseph R., Bonfield W. (1998). Hydroxyapatite-polyethylene composites for bone substitution: Effects of ceramic particle size and morphology. Biomaterials.

[56] Wells J.K., Beaumont P.W.R. (1985). Debonding and pull-out processes in fibrous composites. J. Mater. Sci..

[57] Joseph S., Sreekala M.S., Oommen Z., Koshy P., Thomas S. (2002). A comparison of the mechanical properties of phenol formaldehyde composites reinforced with banana fibres and glass fibres. Compos. Sci. Technol..

[58] Rahman M.R., Huque M.M., Islam M.N., Hasan M. (2009). Mechanical properties of polypropylene composites reinforced with chemically treated abaca. Compos. Part A Appl. Sci. Manuf..

